# Assessing the Feasibility and Acceptability of the Daybreak Drink Tracker: Prospective Observational Study

**DOI:** 10.2196/57403

**Published:** 2024-12-18

**Authors:** Kathryn Fletcher, Dominique Robert-Hendren

**Affiliations:** 1 Hello Sunday Morning, 103 Alexander Street Crows Nest, Sydney, New South Wales, 2065, Australia, 61 1300 403 196; 2 Centre for Mental Health, Swinburne University of Technology, Melbourne, Australia

**Keywords:** app, alcohol use, self-monitoring, tracker, digital health

## Abstract

**Background:**

Excessive alcohol use is associated with significant harms, with wide-ranging social and economic impacts. Efforts to prevent and reduce the harmful use of alcohol are a public health priority. Smartphone apps have the potential to provide accessible and cost-effective support to those seeking to reduce alcohol consumption; however, the evidence base regarding which components are effective is lacking. Self-monitoring is considered as one of the most effective components for behavior change across multiple health domains, yet there is mixed evidence for its role in the alcohol use space. An improved understanding of the use, acceptability, and outcomes of smartphone apps and their inherent components is required to determine their potential role in alcohol behavior change.

**Objective:**

We investigated the feasibility and acceptability of the Drink Tracker, a novel feature of the commercially available Daybreak (Hello Sunday Morning) app.

**Methods:**

The Daybreak app is accessible worldwide via major app stores and is offered free of charge to Australian residents. Individuals (aged over 18 years) registering for Daybreak were invited to access the Drink Tracker to monitor their alcohol consumption as part of an uncontrolled observational prospective study. Feasibility was assessed via uptake and frequency of use of the Drink Tracker. Acceptability was measured via participant feedback to determine overall satisfaction, perceived helpfulness, and likelihood of recommending the Drink Tracker to others. Self-reported changes in alcohol consumption (Alcohol Use Disorders Identification Test score) and psychological distress (Kessler Psychological Distress Scale score) at 3-month follow-up were also measured. Preliminary data collected for the first 4 months (October 2023 to February 2024) of the study were reported, including 3-month follow-up outcomes.

**Results:**

Feasibility was demonstrated, with almost 70% (2847/4119) of those registering for Daybreak going on to access the Drink Tracker. Of those accessing the Drink Tracker, 71.1% (n=2024) consented to research, comprising the final participant sample. Frequency of use was high, with over half of participants (1112/2024, 54.9%) using the Drink Tracker more than once, and more than one-third (757/2024, 37.4%) using the Drink Tracker more than 5 times. Of the 30 participants completing a 3-month follow-up, acceptability was high, with 73% (n=22) reporting high satisfaction levels with the Drink Tracker overall, 87% (n=26) indicating it was easy to use and rating a mean score of 7.7 (SD 2.8) out of 10 in terms of likelihood of recommending to others. Significant reductions in alcohol consumption (*P*<.001) and psychological distress scores (*P*<.001) were observed at the 3-month follow-up.

**Conclusions:**

Our results suggest that the Daybreak Drink Tracker is highly feasible and acceptable in supporting individuals accessing commercially available smartphone apps to change their relationship with alcohol. While positive clinical outcomes were observed, the absence of a control group disallows any conclusions with regard to the efficacy of the Drink Tracker. Further testing via a randomized controlled trial is required.

## Introduction

### Background

Excessive alcohol use is associated with significant harms, contributing to 3 million deaths globally each year, and 5.1% of the global burden of disease and injury [1]. The social and economic impact is widespread, ranging from personal and societal consequences (eg, premature death or medical illness, loss of employment, and loss of social utility use) [2] to costs amounting to an estimated 2.6% of the world’s gross domestic product [3]. Efforts to prevent and reduce the harmful use of alcohol are therefore a public health priority.

Smartphone apps have the potential to overcome barriers to treatment-seeking, facilitate behavior change, and improve health outcomes by providing accessible and cost-effective support on a personal device [4,5]. However, despite a growing number of commercially available alcohol-reduction apps, the majority have little or no evidence base [6]. Efficacy evidence for alcohol-reduction apps is still in its infancy, with mixed results. For example, among those used for patient groups, some apps have shown significant reductions in heavy drinking days [7,8] and increases in days abstinent [9], while others found no significant differences in alcohol consumption outcomes between intervention and control groups [10,11]. In a community sample, a quasi-experimental randomized controlled trial (RCT) of the Daybreak app reported significant improvements in alcohol consumption at 3 months [12]. More recently, hazardous drinkers in an undergraduate sample of university students significantly reduced their alcohol intake compared to those in the control condition, following the use of a brief electronic intervention (combining a smartphone app alcohol tracker with a web-based intervention) designed to provide personalized feedback [13].

As overviewed by Crane et al [6], while many alcohol-reduction apps are available, little is known about which specific features effectively promote behavior change. One way of looking at potentially active ingredients is to assess the behavior change techniques (BCTs) that apps contain. A BCT is “an observable, replicable, and irreducible component of an intervention designed to alter or redirect causal processes that regulate behaviour; that is, a technique is proposed to be an ‘active ingredient’ (e.g., feedback, self-monitoring, and reinforcement)” [14]. Evidence suggests one such BCT—self-monitoring—results in more significant behavior change and promotes goal attainment [15]. Self-monitoring is the act of noticing and recording goal-related behavior [16]. It often involves self-evaluation, periodic measurement, and recording of the target behavior by the individual, allowing tracking of behavior over time. Self-monitoring is the most widely used technique in behavior change interventions seeking to promote health and wellness [17]. Self-monitoring is a theoretically and empirically grounded strategy [18]. Social cognitive theory posits that self-monitoring influences an individual’s motivations and actions by increasing their attention toward their behavior and is therefore considered to be a crucial element of behavior change [19]. In general, tools that help individuals monitor their goal progress are more effective at promoting behavior change [20]. By prompting monitoring, the individual is more likely to attain their goals, and the more frequent the monitoring, the higher the chance of success. Qualitative findings suggest self-monitoring can raise consciousness and encourage the individual to reflect on their behavior [18]. This self-reflection provides intrinsic motivation and long-term behavior change, in line with the transtheoretical model of change [21]. Further, self-monitoring increases accountability and intrapersonal competition and helps individuals to assume responsibility for their behaviors [18]. Digital self-monitoring is particularly well-placed support to health-related self-management, offering flexible use in everyday life [22].

Within the alcohol behavior change context, self-monitoring can take different forms, from a simple recording of alcohol-free versus drinking days to detailed feedback and recording of different aspects associated with drinking. For example, the Drink Less app contains “Self-monitoring and Feedback” features which allow participants to record their alcohol consumption and provide feedback on their consumption and the consequences of consumption (calories consumed, money spent and effect on mood, productivity, and sleep), as well as progress against goals [23].

### Does Self-Monitoring Alcohol Consumption Work?

As overviewed by Carpenter et al [24], systematic reviews indicate that self-monitoring is one of the most effective intervention components for behavior change across multiple health domains. However, evidence is mixed for the role of self-monitoring in alcohol behavior change. Within digital health interventions, self-monitoring with feedback was found to increase engagement by app end-users and was rated positively in terms of helpfulness, satisfaction, and recommendation to others [25]. By contrast, in a meta-regression of 41 RCTs of alcohol reduction digital behavior change interventions, Garnett et al [15] found BCTs other than self-monitoring (eg, “behavior substitution,” “problem-solving,” and “credible source”) were associated with greater alcohol reduction. However, it was noted that self-monitoring was used less frequently in the included studies (with only one-quarter of individuals asked to self-monitor their drinking despite the ability of digital health interventions to facilitate this) despite good evidence of its effectiveness in other behavior change domains. Self-monitoring is recommended by clinical guidelines as an effective component of interventions for alcohol reduction and other health-related behaviors [26]. Furthermore, international experts in the field of alcohol behavior change have ranked self-monitoring as one of the top techniques most likely to be effective for reducing alcohol use [27]. Taken together, a range of BCTs including self-monitoring are likely to be effective in reducing alcohol consumption. However, prior to establishing the effectiveness of app components such as self-monitoring, an important first step is to understand how such components are used (feasibility: initial uptake, ongoing use, etc), and whether they meet the needs of the end user (acceptability: satisfaction, perceived use, etc). Feasibility and acceptability data provide context for why certain components may be effective, assisting in the interpretation of subsequent outcomes and informing ongoing intervention development.

### This Study

Daybreak, first launched in 2016 by Hello Sunday Morning, is a smartphone app aiming to assist individuals in changing their relationship with alcohol. Daybreak is accessible worldwide via major app stores, supported by the Australian Government, and is offered free of charge to Australian residents. Daybreak currently has more than 900 new members registering each month on average, potentially reaching over 10,800 individual new members seeking to improve their relationship with alcohol each year. Daybreak can be conceptualized as an evidence-based mobile health (mHealth) tool that is highly accessible, overcomes common barriers to treatment-seeking (stigma, cost, and flexibility), and has the potential to streamline treatment by empowering individuals at whatever stage they are to take the next step on their alcohol behavior change journey [12]. Daybreak features currently include (1) clinically validated tools assessing alcohol use and psychological well-being, providing feedback to members along with service and resource information; (2) a moderated peer-led community offering emotional and practical support; and (3) behavioral experiments drawing from evidence-based psychological therapies. The Drink Tracker feature was introduced into Daybreak in October 2023 to support members in setting goals and tracking their alcohol consumption over time.

### Objectives

The overarching aim of this study is to assess the feasibility and acceptability of the Drink Tracker component of the Daybreak app. Feasibility was defined in terms of uptake (ie, the proportion of members registering for Daybreak who go on to access the Drink Tracker, with over 50% considered as “high” uptake) and frequency of use of the Drink Tracker (ie, using the Drink Tracker more than once). Acceptability was measured via feedback from participants in terms of their overall satisfaction, perceived helpfulness, and likelihood of recommending the Drink Tracker to others at a 3-month follow-up. Finally, we examined changes in alcohol consumption and psychological well-being, assessed via self-report at 3-month follow-up.

## Methods

### Design

Individuals (aged 18 years and over) registering for Daybreak were invited to access the Drink Tracker to monitor their alcohol consumption as part of an uncontrolled observational prospective study. Marketing and advertising for Daybreak included social media posts (Facebook and Instagram), Google Ads keywords, and information in the Hello Sunday Morning newsletter. When registering, individuals completed basic demographics questions and 2 validated measures of alcohol consumption and well-being. Those consenting to participate in the research were additionally asked to complete (1) a feedback questionnaire on their experience of the Drink Tracker at 3 months and (2) follow-up measures of alcohol consumption and well-being. Reminders (ie, push notifications) to complete Drink Tracker check-ins were sent every month (4 in total during the study period) to prompt ongoing engagement. This paper reports preliminary data collected for the first 4 months (October 2023 to February 2024).

### Participants

Individuals were eligible to participate in the Drink Tracker research study if (1) they were aged 18 years or older and (2) completed registration for Daybreak.

### Intervention

Basic demographic questions (age, gender, and country of residence), drinking goal selection, and validated questionnaires assessing baseline (1) alcohol consumption via the Alcohol Use Disorders Identification Test (AUDIT) [28] and (2) psychological distress via the Kessler Psychological Distress Scale (K10) [29] are completed as part of Daybreak registration, and monthly thereafter. Brief feedback on AUDIT and K10 results are provided (ie, risk levels, along with information for support and resources). The Drink Tracker is then introduced as a tool to monitor alcohol consumption. The Drink Tracker allows participants to track (1) alcohol-free days, (2) drinking days, and (3) the standard number of drinks consumed on a drinking day. Participants nominate Drink Tracker goals and can choose to share their goals with the Daybreak peer-led community. Drink Tracker results are displayed via calendar and graphs to allow participants to visually track their progress over time (see Multimedia Appendix 1). As part of onboarding messaging, participants are encouraged to check in daily to track their alcohol consumption, and each completed check-in is followed by a congratulatory message. Finally, editing functionality is provided for participants to edit their Drink Tracker goals over time and update check-ins.

### Measures

#### AUDIT

The 10-item AUDIT [28] assesses the risk of alcohol-related harm across 3 domains: alcohol consumption, dependence, and alcohol-related consequences. Scores range from 0 to 40, with higher scores indicating a greater likelihood of hazardous and harmful drinking (0‐7=low risk for alcohol dependence, 8‐15=moderate risk for alcohol dependence, 16‐19=high risk for alcohol dependence, 20‐40=very high risk or possible alcohol dependence) [28]. As the AUDIT is based on a 12-month reference period, the Alcohol Use Disorder Identification Test Consumption subscale (AUDIT-C) [30], comprising the first 3 questions of the AUDIT, is recommended as the outcome measure to assess changes over time. Previous research has shown that the AUDIT-C can predict clinical outcomes at 12 months [31].

#### K10

The K10 is a widely used measure of psychological distress and is recommended as the screening measure for comorbid mental health conditions in individuals presenting with alcohol use disorder [32]. Total severity scores range from 10 to 50, with higher scores indicating greater levels of psychological distress (10‐15=low distress, 16‐21=moderate distress, 22‐29=distress, ≥30=severe mental health distress) [33]. The temporal stability of the K10 in treatment- and non–treatment-seeking samples has recently been confirmed [34], supporting its use as an outcome measure. The K10 has demonstrated strong psychometric properties and can be used as a valid predictor of mental health, mood, and anxiety disorders [34-36].

#### Feedback and Follow-Ups

Feedback questions assessed (1) satisfaction with the Drink Tracker and its components and likelihood of recommending the tool to others and (2) a series of brief open-ended questions asking about aspects of the Drink Tracker that were liked or least liked, areas for improvement, advantages of tracking and any changes made or learnings since using the Drink Tracker. Monthly follow-ups comprised the AUDIT-C and K10. This paper reports on 3-month outcomes.

### Statistical Analyses

Statistical analyses were conducted using SPSS Statistics for Windows (version 29.0.2.0; IBM Corp). Descriptive statistics were used to quantify respondent characteristics. Student 2-tailed *t* tests were used to compare continuous variables, whereas the Pearson *χ*^2^ test was used to compare categorical variables between 2 groups. Inductive thematic analysis was conducted with open-ended questions in the follow-up survey, following principles outlined by Braun and Clarke [37]. The first author (KF) familiarized herself with the data set by reading, rereading, and noting preliminary codes related to the study objective. Codes were then grouped into provisional themes using Microsoft Excel. Coding anomalies and provisional themes were then discussed and agreed upon by both authors, and themes were considered final if they captured the perspectives of multiple participants and were grounded in the data.

Paired samples *t* tests were used to analyze the changes in outcomes on AUDIT and K10 from baseline to 3 months. Bonferroni correction was applied to account for multiple comparisons. An a priori power analysis was conducted for outcomes at 3 months using G*Power [38] to determine the minimum sample size. The results indicated that the required sample size to achieve 80% power at a significance criterion of .05 was 27 for a paired samples *t* test.

### Ethical Considerations

The study protocol and consent procedures were approved by the Bellberry Human Research Ethics Committee (2023-06-736). Study participants provided informed consent within the Daybreak app and were advised that participation was voluntary, they were free to withdraw at any time, and their data would be stored securely and anonymously.

## Results

### Overview

Of the 4119 individuals completing registration for Daybreak during the 4-month study period, 2847 (69.1%) accessed the Drink Tracker. Of those accessing the Drink Tracker, 71.1% (n=2024) consented to Drink Tracker research, comprising the final participant sample for the study. The characteristics of the study participants are presented in Table 1.

**Table 1.** Participant characteristics (N=2024).

Characteristics		Values
Female, n (%)		1288 (63.6)
Age (years), mean (SD)		43.1 (11)
**Country of residence, n (%)**		
	Australia	1788 (88.3)
	United States	79 (3.9)
	United Kingdom	24 (1.2)
	Canada	22 (1.1)
	Ireland	13 (0.6)
	New Zealand	13 (0.6)
	South Africa	9 (0.4)
	Other	76 (3.8)
**Alcohol use**		
	AUDIT^a^ score, mean (SD)	20.1 (7.8)
	Low risk, n (%)	109 (5.4)
	Moderate risk, n (%)	495 (24.5)
	High risk, n (%)	340 (16.8)
	Very high risk, n (%)	1080 (53.4)
	AUDIT-C^b^ score, mean (SD)	8.4 (2.5)
**Psychological distress, n (%)**		
	K10^c^ score, mean (SD)	25 (8.7)
	Low distress, n (%)	397 (15.2)
	Moderate distress, n (%)	491 (24.3)
	High distress, n (%)	620 (30.6)
	Very high distress, n (%)	606 (29.9)
**Frequency of Drink Tracker check-ins, n (%)**		
	Once	583 (28.8)
	Twice	165 (8.2)
	3‐5 times	190 (9.4)
	More than 5 times	757 (37.4)
	Never	329 (16.3)

a AUDIT: Alcohol Use Disorders Identification Test.

b AUDIT-C: Alcohol Use Disorder Identification Test Consumption subscale.

c K10: Kessler Psychological Distress Scale.

Women were overrepresented, as were those residing in Australia. The average age of the sample was 43.1 years, ranging from 18 to 79 years. AUDIT scores indicated that 70.2% (1420/2024) of the sample were at a “high” or “very high” risk of alcohol dependence. K10 scores indicated that 60.5% (1226/2024) of the sample were experiencing “high” or “very high” levels of psychological distress. In terms of frequency of use of the Drink Tracker during the 4-month study period, 54.9% (1112/2024) accessed the Drink Tracker more than once.

### Feedback and Follow-Up Data

Of the 544 participants who had been part of the study for at least 3 months (and were therefore sent the 3-month follow-up surveys for completion), 30 (5.5%) completed the questionnaires (feedback questions, AUDIT-C, and K10). Of these, 14 (46.7%) were female, with an average age of 47.5 (SD 10.3, range 26‐66) years, and 30 (100%) were residing in Australia. Compared to the baseline participant sample (ie, all participants who consented to research and accessed the Drink Tracker, irrespective of how long they had been in the study), no significant differences were observed in the follow-up group in terms of gender (*χ*^2_2_^=4.6; *P*=.1), baseline AUDIT (mean 20.1, SD 7.8 vs mean 18.7, SD 7.5; *t*_2022_=1; *P*=.34) and AUDIT-C scores (mean 8.4, SD 2.5 vs mean 8.4, SD 2.6; *t*_2022_=0; *P*=.98), or K10 scores (mean 25, SD 8.7 vs mean 24.5, SD 7.9, *t*_2022_=0.3; *P*=.74). However, the mean age of the follow-up group was significantly higher (mean 43.1, SD 11 vs mean 47.5, SD 10.3; *t*_2022_=−2.2; *P*=.03).

In terms of feedback, 73% (22/30) indicated they were “satisfied or very satisfied” with the Drink Tracker overall. Participants were asked how likely they would be to recommend the Drink Tracker to others (0=very unlikely to recommend to 10=very likely to recommend), rating a mean score of 7.7 (SD 2.8).

Participants were presented with a series of statements with regard to aspects of the Drink Tracker and asked about the extent to which they agreed (5-point scale; strongly disagree to strongly agree) with each statement (Table 2).

Ease of use, helping to keep on track with drinking goals, increased accountability, and improved understanding of drinking patterns were the most appreciated aspects of the Drink Tracker.

**Table 2.** Agreement levels (agree or strongly agree) in relation to Drink Tracker aspects (n=30).

	Participant responses, n (%)
The Drink Tracker was easy to use	26 (86.7)
The Drink Tracker feedback (calendar, graph) was useful	20 (66.7)
The Drink Tracker helped me keep on track with my drinking goals	23 (76.7)
The Drink Tracker was motivating	20 (66.7)
The Drink Tracker helped keep me accountable	22 (73.3)
The Drink Tracker improved my understanding of my drinking patterns	22 (73.3)

In terms of themes from open-ended responses, the “most liked” aspect of the Drink Tracker was its simplicity and ease of use, along with the visual representation of results (eg, seeing alcohol-free days build up over time). “Least liked” aspects included an inability to backdate, with participants wanting the option to record drinking patterns prior to the release of the Drink Tracker in October 2023. Themes in relation to recommendations for improvements included the ability to reset start-dates, backdate, and added functionality (eg, display cumulative health and financial costs associated with drinking, targeting the app more toward goals, and reminders to track). The advantages of tracking included increased awareness of drinking habits, feeling more motivated to reduce consumption (and a sense of achievement from seeing success), and experiencing success in reducing or stopping drinking altogether. Many participants (18/30, 60%) indicated that they had successfully reduced or stopped drinking since using the Drink Tracker. Finally, learnings arising from the use of the Drink Tracker included better awareness of drinking habits, realization that it was possible to drink less or stop drinking, and feeling less alone and more supported on the alcohol behavior change journey along with other Daybreak members.

Turning to AUDIT-C and K10 results, paired samples *t* tests showed significant within-group reductions from baseline to 3-month follow-up AUDIT-C scores (mean 8.3, SD 2.6 vs mean 4, SD 4.3, *t*_29_=4.6; *P*<.001; Cohen *d*=0.83, 95% CI 0.41‐1.24) and K10 scores (mean 24.5, SD 7.9 vs mean 18.7, SD 7.5, *t*_29_=4.2; *P*<.001; Cohen *d*=0.77, 95% CI 0.36‐1.18).

## Discussion

### Principal Findings

This study investigated the feasibility and acceptability of the Drink Tracker, along with self-reported improvements in alcohol consumption and psychological distress scores at 3-month follow-up. Key findings are now summarized.

Feasibility was demonstrated, with the majority (2847/4119, 69.1%) of individuals registering for Daybreak subsequently accessing the Drink Tracker during the study period. High uptake aligns with prior research reporting that prospective alcohol app users value the self-monitoring support that an app can provide [39,40]. In terms of frequency of use, 54.9% (1112/2024) used the Drink Tracker more than once, and more than one-third (757/2024, 37.4%) used the Drink Tracker more than 5 times. This compares favorably to a previous study reporting usage rates of 20.5% (self-reported use of >1 time) and 1.8% (self-reported use of >5 times) of a self-monitoring component within a similar alcohol reduction app [41]; however, two key differences in our study were (1) the use of app data in-the-moment rather than self-reported data from a questionnaire at follow-up for self-monitoring frequency and (2) regular push notifications to prompt app usage. As noted by Bertholet et al [41], app developments in the alcohol context should focus on dosage and delivery of prompts that increase use (eg, reminder messages and push notifications), given the high attrition rates associated with mHealth apps [42].

Turning to follow-up data, the Drink Tracker was favorably rated, with the majority (22/30, 73%) indicating they were “satisfied or highly satisfied” overall and indicating a high likelihood to recommend the tool to others. Aspects of the Drink Tracker that were most appreciated (with over 70% indicating they “agreed or strongly agreed”) included its ease of use, while keeping them on track with their drinking goals, increasing accountability, and improving their understanding of drinking patterns. These results are supported by social cognitive theory and the transtheoretical model of change, whereby self-monitoring can influence motivations and actions [19,21]—in this case through increasing accountability toward changing alcohol use behavior and improving understanding of usage patterns.

Open-ended responses highlighted an appreciation for the visual representation of results (with this feedback supporting motivation and a sense of achievement); however, the inability to backdate for longer periods of time was nominated as a key feature for improvement. These align with recommendations provided by users of similar commercially available apps (eg, Drinkaware), whereby the ability to view changes in alcohol consumption over time was highlighted as important, including clear visual summaries of longer-term drinking trends [43].

For those completing 3-month follow-up AUDIT-C and K10 measures, self-reported scores reduced significantly for both alcohol consumption and psychological distress. Given the mixed evidence for app effectiveness in reducing alcohol consumption [44], this is an encouraging finding. Similar findings were reported by those using the Drinkaware app, with self-reported alcohol consumption levels reducing within the first week and then plateauing by week 12 [43]. The longer-term impact of apps such as Daybreak on alcohol consumption is an important area for future investigation.

### Strengths and Limitations

The study had several strengths and limitations. Strengths include a large sample of individuals accessing a commercially available app. These individuals are representative of those in the real-world context seeking to reduce alcohol consumption on their own using behavior change apps, as opposed to individuals specifically recruited for a research study. Unlike many commercially available apps, Daybreak incorporates evidence-based components, offering a strong foundation from which to test novel features. Building on this foundation, high uptake of the new Drink Tracker feature was observed, frequency of use was relatively high, and participants provided positive feedback. Taken together, study findings contribute to the urgent need for an improved understanding of the use, acceptability, and outcomes of smartphone apps—and their components—for alcohol use [41].

Regarding this study’s limitations, only 5.5% (30/544) completed the 3-month follow-up questionnaires. While this group was comparable to those accessing the Drink Tracker at baseline in terms of gender, alcohol use, and psychological distress scores (albeit being significantly older), these participants likely comprise a highly engaged group that may not be representative of all individuals using mHealth apps more broadly, and who are willing to engage in research. The 514 participants who did not complete the 3-month follow-up questionnaires were not contacted, limiting conclusions that can be drawn and highlighting the need for assertive follow-up to ensure participant experiences are generalizable. High attrition rates are commonly associated with mHealth apps, with estimates ranging from 38% to 84% in app-based intervention studies [42]. Indeed, one in five end users abandon commercial apps in real-world settings (such as Daybreak) after the first use [45]. While push notifications were sent to participants to improve engagement with Daybreak more broadly, follow-up questionnaires were set to expire after 7 days, and no reminders were sent to complete questionnaires. Therefore, individuals not using the app on a regular basis would have been lost to follow-up. To improve ongoing engagement, user needs and preferences in app development processes are indicated to keep individuals engaged [46], in addition to more rigorous follow-up for completion of research questionnaires (eg, multiple reminders). We have previously reported a 3-month follow-up questionnaire completion rate of 17.6% for a publicly available web-based screening tool recruiting a similar sample of adults seeking to reduce alcohol consumption [47]. Although not directly comparable given the (1) differing technology (web-based tool vs app), (2) use of reminders (email), and (3) offering of financial incentives in our previous study, high attrition rates from follow-ups in this population remain a key challenge for those seeking to evaluate the impact of digital health interventions on alcohol behavior change. Nonetheless, apps such as Daybreak still have the potential to positively impact public health so long as engagement strategies are optimized to promote reduced alcohol consumption. Other limitations include the reliance on self-reported outcomes, which are subject to social desirability or recall bias. Further, associations between Drink Tracker usage patterns and 3-month outcomes were not examined in this study; therefore, we were unable to determine the impact of adherence to self-monitoring (eg, frequency and consistency of use) on participant outcomes. Greater self-monitoring adherence has been associated with positive behavior change in the weight loss literature [48], while self-reported use of an app (containing a self-monitoring feature among other components) more than once was associated with less weekly alcohol consumption at 3-month follow-up [41]. Adherence to self-monitoring tools and their impact on outcomes is an important area for future investigation.

Finally, while self-reported alcohol consumption reduced and psychological well-being improved, the lack of a control group disallows any conclusions to be drawn regarding efficacy. The Drink Tracker is one of several components of Daybreak; therefore, disentangling the impact of the Drink Tracker on outcomes from other features (eg, support from the peer-led community) was not possible in this study. Improvements may also be explained by regression to the mean. As noted by Crane et al [24], motivation to use alcohol reduction apps may be high when an individual is drinking excessively; therefore, regression to the mean in this context suggests that consumption scores differing substantially from the true mean tend to be followed by scores closer to the true mean [49], explaining changes over time. Nonetheless, study findings are promising and will inform the development of an RCT to determine how and under what conditions the Drink Tracker may best support those seeking to reduce their alcohol consumption.

### Conclusions

The Daybreak Drink Tracker is a highly feasible and acceptable tool to assist individuals in changing their relationship with alcohol. Further testing via an RCT is required to determine which features (either alone or in combination) of Daybreak effectively promote behavior change and the context within which the Drink Tracker feature is likely to be specifically effective.

## Supplementary material

10.2196/57403 Multimedia Appendix 1 Drink Tracker.

## Abbreviations

AUDIT: Alcohol Use Disorder Identification Test
AUDIT-C: Alcohol Use Disorder Identification Test Consumption subscale
BCT: behavior change technique
K10: Kessler Psychological Distress Scale
mHealth: mobile health
RCT: randomized controlled trial
