# Supplementary figures and images for: Assessing the Feasibility and Acceptability of the Daybreak Drink Tracker: Prospective Observational Study
Source: JMIR Form Res. 2024 Dec 18;8:e57403. doi: 10.2196/57403 (PMC11669369; doi:10.2196/57403)

**Multimedia Appendix 1: Drink Tracker**


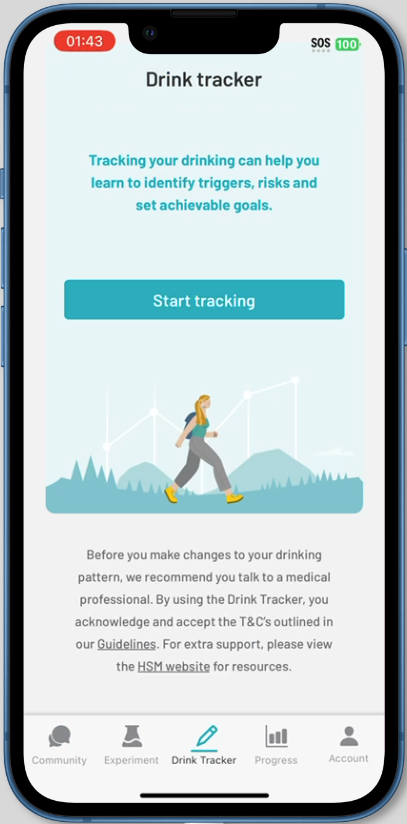

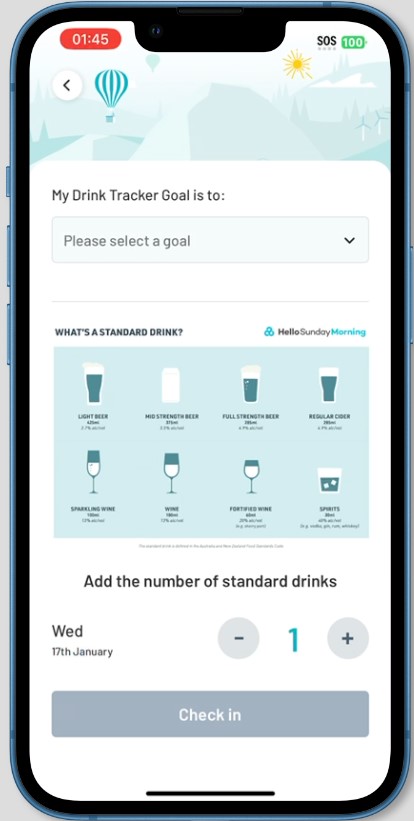


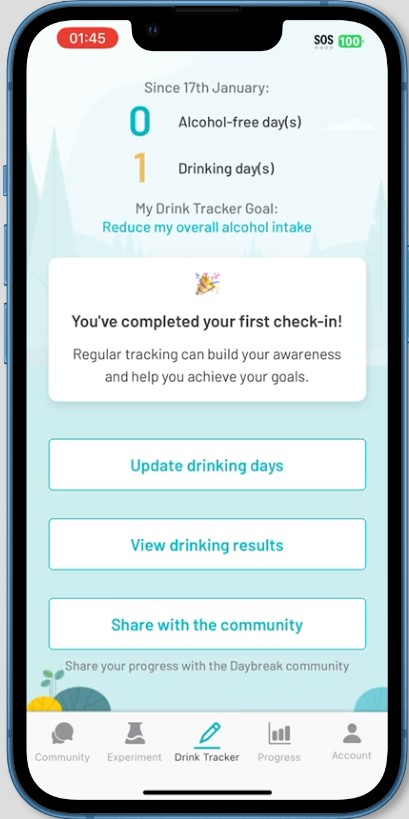

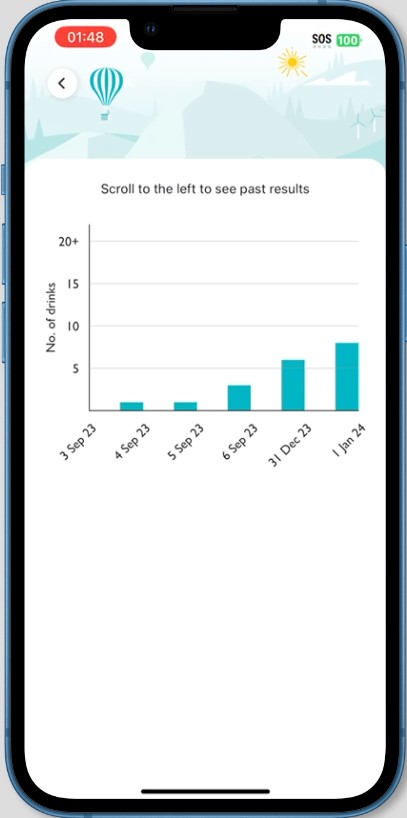

Supplement: Multimedia Appendix 1 [file formative-v8-e57403-s001.docx]
